# Cyclic-di-GMP stimulates keratinocyte innate immune responses and attenuates methicillin-resistant *Staphylococcus aureus* colonization in a murine skin wound infection model

**DOI:** 10.1186/s12866-022-02583-1

**Published:** 2022-07-08

**Authors:** Shuai Gao, Abidullah Khan, Xuhong Chen, Guohui Xiao, Stijn van der Veen, Yin Chen, Xu’ai Lin

**Affiliations:** 1 Department of Medical Microbiology and Parasitology, and Department of Infection of the Children’s Hospital, National Clinical Research Center for Child Health, School of Medicine, Zhejiang University, 866Yuhangtang Road, West Lake District, Hangzhou, 310058 China; 2 Department of Burns, Second Affiliated Hospital, School of Medicine, Zhejiang University, Hangzhou, China; 3 Department of Microbiology, and Department of Dermatology of Sir Run Run Shaw Hospital, School of Medicine, Zhejiang University, Hangzhou, China; 4 Key Laboratory of Emergency Detection for Public Health of Zhejiang Province, Zhejiang Provincial Center for Disease Control and Prevention, 3399 Binsheng Road, Binjiang District, Hangzhou, 310051 China

**Keywords:** MRSA, Skin infection, c-di-GMP, Immunomodulatory

## Abstract

**Background:**

*Staphylococcus aureus* is a leading cause for morbidity and mortality associated with skin and burn wound infections. Therapeutic options for methicillin-resistant *S. aureus* (MRSA) have dwindled and therefore alternative treatments are urgently needed. In this study, the immuno-stimulating and anti-MRSA effects of cyclic di-guanosine monophosphate (c-di-GMP), a uniquely bacterial second messenger and immuno-modulator, were investigated in HaCaT human epidermal keratinocytes and a murine skin wound infection model.

**Results:**

Stimulation of HaCaT cells with 125 μM c-di-GMP for 12 h prior to MRSA challenge resulted in a 20-fold reduction in bacterial colonization compared with untreated control cells, which was not the result of a direct c-di-GMP toxic effect, since bacterial viability was not affected by this dose in the absence of HaCaT cells. C-di-GMP-stimulated or MRSA-challenged HaCaT cells displayed enhanced secretion of the antimicrobial peptides human β-defensin 1 (hBD-1), hBD-2, hBD-3 and LL-37, but for hBD1 and LL-37 the responses were additive in a c-di-GMP-dose-dependent manner. Secretion of the chemokines CXCL1 and CXCL8 was also elevated after stimulation of HaCaT cells with lower c-di-GMP doses and peaked at a dose of 5 μM. Finally, pre-treatment of mice with a 200 nmol dose of c-di-GMP 24 h before a challenge with MRSA in skin wound infection model resulted in a major reduction (up to 1,100-fold by day 2) in bacterial CFU counts recovered from challenged skin tissue sections compared PBS-treated control animals. Tissue sections displayed inflammatory cell infiltration and enhanced neutrophil influx in the c-di-GMP pre-treated animals, which might account for the reduced ability of MRSA to colonize c-di-GMP pre-treated mice.

**Conclusions:**

These results demonstrate that c-di-GMP is a potent immuno-modulator that can stimulate anti-MRSA immune responses in vivo and might therefore be a suitable alternative prophylactic or therapeutic agent for MRSA skin or burn wound infections.

## Background

*Staphylococcus aureus* is an important bacterial pathogen that causes a wide variety of infections and it is globally the most common causative agent of skin, soft tissue and burn-wound infections [1, 2], and the leading cause of death in burn-associated invasive infections [3, 4]. Effective antimicrobial therapies are essential for the management, treatment and prevention of *S. aureus* burn-wound infections. However, the emergence and spreading of methicillin-resistant *S. aureus* (MRSA) over the past decades have made effective management and therapy of *S. aureus* infections increasingly problematic [5, 6], while MRSA is now accounting for more than half of the burn wound infections reported by burn units [4]. Vancomycin has become the recommended first-line therapy for MRSA infections, however, vancomycin resistance has already developed [7, 8] and vancomycin-resistant *S. aureus* (VRSA) strains have been encountered in a number of countries [9–11]. Alternative therapies for MRSA include the oxazolidinone linezolid, the cyclic lipopeptide daptomycin and the cephalosporin ceftaroline, but resistance against these antimicrobials has already been established [12]. Therefore, there is an urgent need to develop novel antimicrobials [13] and/or alternative strategies to ensure future treatment of MRSA remains available [14].

An alternative strategy to target or prevent MRSA infections in skin and burn wounds might be the topical or systemic administration of cyclic di-guanosine monophosphate (c-di-GMP), a prokaryote-specific second messenger that it essential for signaling in a wide variety of bacterial cues and therefore a central regulator in bacterial physiology and pathogenesis [15, 16]. It has already been shown that c-di-GMP regulates bacterial motility, biofilm formation, quorum sensing, cell cycle and differentiation, adhesion and host colonization [15, 17]. Furthermore, due to its prokaryotic specificity, host cells have evolved to sense c-di-GMP as a danger molecule. It is specifically recognized in the host cell cytosol by stimulator of interferon genes (STING), which induces a type 1 interferon (IFN) response [18, 19], and by the NLRP3 inflammasome, which induces an interleukin (IL)-1β response [20]. In addition, c-di-GMP injection results in adjuvant-like responses that include immune cell recruitment, dendritic cell (DC) activation and maturation, induction of cytokine and chemokine levels, and T cell stimulation [21]. These results indicate that c-di-GMP may be a suitable therapeutic that stimulates the host to build an antimicrobial immune response and alleviates the burden on antibiotics and the concomitant rising antimicrobial resistance.

It has been demonstrated for *S. aureus* that addition of exogenous c-di-GMP in vitro inhibits bacterial interactions and biofilm formation [22] and in vivo inhibits colonization when provided either as a therapeutic or prophylactic in a mastitis mouse model of infection [21, 23, 24]. Furthermore, immunization of mice with c-di-GMP-adjuvanted nontoxic mutant staphylococcal enterotoxin C (mSEC) or clumping factor A (ClfA) resulted in protective immune responses against a subsequent *S. aureus* challenge [24]. Similarly, prophylactic administration of c-di-GMP provided protection in a mouse model of infection against subsequent challenges by *Acinetobacter baumannii* [25], *Bordetella pertussis* [26], *Klebsiella pneumoniae* [27] and *Streptococcus pneumoniae* [28], with mice showing enhanced recruitment of immune cells, enhanced cytokine and chemokine production levels and immune cells with increased nitric oxide levels. In the current study, c-di-GMP was explored for its immunostimulatory properties in HaCaT human epidermal keratinocyte cells and its antimicrobial properties against an MRSA challenge in a murine skin wound model of infection.

## Results

### C-di-GMP inhibits MRSA colonization of HaCaT cells

**Fig. 1:**
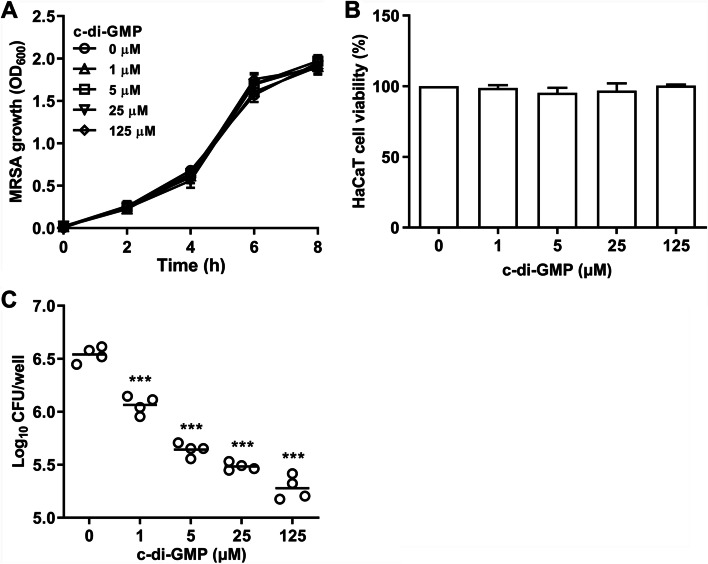
Stimulation of HaCaT cells with c-di-GMP inhibits MRSA colonization. **A** MRSA strain HRCH2019-1 was grown in TSB at 37 °C and 200 rpm in the presence of c-di-GMP (0, 1, 5, 25, or 125 μM) and bacterial growth was monitored by absorbance measurements (OD_600_) at 2-h intervals. **B** Cytotoxicity of c-di-GMP against HaCaT cells was determined with the CCK-8 assay. Viability of HaCaT cells after treatment for 24 h with c-di-GMP at 1, 5, 25 and 125 μM was determined relative to untreated control cells. **C** HaCaT cells were incubated for 12 h with c-di-GMP at 0, 1, 5, 25, or 125 μM and challenged with MRSA strain HRCH2019-1. Bacterial colonization of HaCaT cells was determined after 3 h incubation. Significant differences compared with 0 μM c-di-GMP were identified by one-way ANOVA with Dunnett’s multiple comparisons test (GraphPad Prism). ***, *P* < 0.001

C-di-GMP does not display direct antimicrobial properties against MRSA strain HRSH2019-1, since addition of c-di-GMP to bacterial cultures did not affect growth curves (Fig. 1A). To investigate whether c-di-GMP may be a suitable antimicrobial immune response modulator against MRSA during skin or burn-wound infections, its cytotoxicity was first analyzed using the HaCaT human epidermal keratinocyte cell line. C-di-GMP was added to the HaCaT cells at concentrations ranging from 1 to 125 μM and cytotoxicity was analyzed using the CCK-8 cell viability assay. Even at the highest c-di-GMP concentration, no reduction in cell viability was observed compared with untreated control cells (Fig. 1B), indicating that c-di-GMP does not display cytotoxicity. HaCaT cells were subsequently stimulated with c-di-GMP for 12 h and challenged with MRSA strain HRCH2019-1. Our results showed that colonization of the HaCaT cells was significantly reduced in a c-di-GMP-concentration-dependent manner (Fig. 1C). At the highest c-di-GMP concentration of 125 μM, association of MRSA with HaCaT cells was approximately 20-fold lower compared with untreated control cells. Therefore, these results indicate that c-di-GMP likely induced an anti-MRSA response in the HaCaT cells.

### C-di-GMP induces secretion of AMPs and chemokines in HaCaT cells

**Fig. 2:**
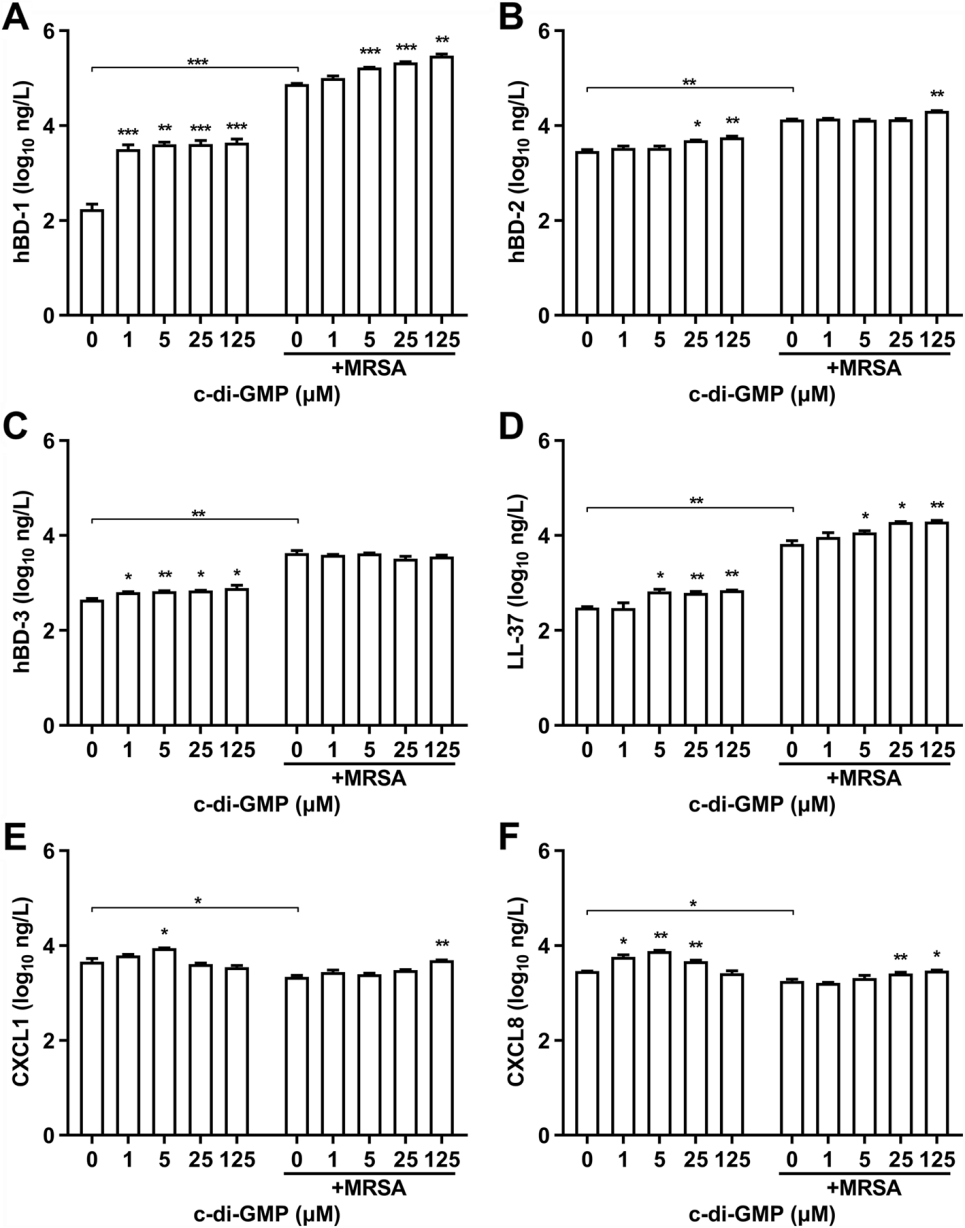
Stimulation of HaCaT cells with c-di-GMP induces secretion of antimicrobial peptides and chemokines. HaCaT cells were incubated for 12 h with c-di-GMP at 0, 1, 5, 25, or 125 μM and challenged with MRSA strain HRCH2019-1 for 3 h. Culture medium of infected and uninfected control cells was harvested for quantification of antimicrobial peptides (AMPs) and chemokines by ELISA. **A** Quantification of human AMP β-defensin 1 (hBD-1). **B** Quantification of AMP hBD-2. **C** Quantification of AMP hBD-3. **D** Quantification of AMP LL-37. **E** Quantification of chemokine CXCL1. **F** Quantification of chemokine CXCL8. Significant differences compared with 0 μM c-di-GMP for both the MRSA-challenged and unchallenged cells and for other specified comparisons were identified by two-way ANOVA with Dunnett’s multiple comparisons test (GraphPad Prism). *, *P* < 0.05; **, *P* < 0.01; ***, *P* < 0.001

Keratinocytes have been established to mount a rapid antimicrobial response against *S. aureus* and other pathogens frequented on the skin upon direct contact, with antimicrobial responses consisting of AMPs for direct microbicidal activity and chemokines for recruitment of phagocytes [29]. Therefore, secretion of antimicrobial peptides and chemokines by HaCaT cells upon stimulation by c-di-GMP and challenge by MRSA strain HRCH2019-1 was investigated by ELISA. Stimulation of HaCaT cells with c-di-GMP significantly enhanced secretion of all four tested AMPs hBD-1, hBD-2, hBD-3 and LL-37 compared with the 0 μM unstimulated control (Fig. 2A-D). Induction of hBD-1 secretion was particularly elevated at 18- to 25-fold, while enhanced secretion of hBD-2 and LL-37 was only apparent at higher c-di-GMP concentrations. Challenging of HaCaT cells with MRSA strain HRCH2019-1 in the absence of c-di-GMP (0 μM c-di-GMP control) also resulted in significantly elevated secretion of the AMPs, with induction of hBD-1 at 400-fold being most pronounced. Importantly, MRSA challenging of c-di-GMP-stimulated HaCaT cells resulted in further elevated secretion of hBD-1 and LL-37 in a c-di-GMP-concentration-dependent manner, indicating that responses were additive. In contrast, this effect was not observed for hBD-2 and hBD-3, which secretion appeared largely dependent on MRSA challenge. Neutrophils play an essential role in targeting and eliminating *S. aureus* during a skin infection [30, 31], while keratinocytes are able to secrete CXCL1 and CXCL8, which are the primary chemokines responsible for neutrophil recruitment and stimulation of neutrophil degranulation [31–33]. HaCaT cell secretion of CXCL1 and CXCL8 was significantly enhanced by stimulation with c-di-GMP, but enhanced secretion was only observed at the lower c-di-GMP concentrations and peaked at 5 μM (Fig. 2E-F). Challenging of HaCaT cells with MRSA strain HRCH2019-1 in the absence of c-di-GMP stimulation actually resulted in a significant decrease of CXCL1 and CXCL8 secretion, which was reversed to control levels at the highest c-di-GMP concentrations.

### C-di-GMP induces MHC gene expression in HaCaT cells

**Fig. 3:**
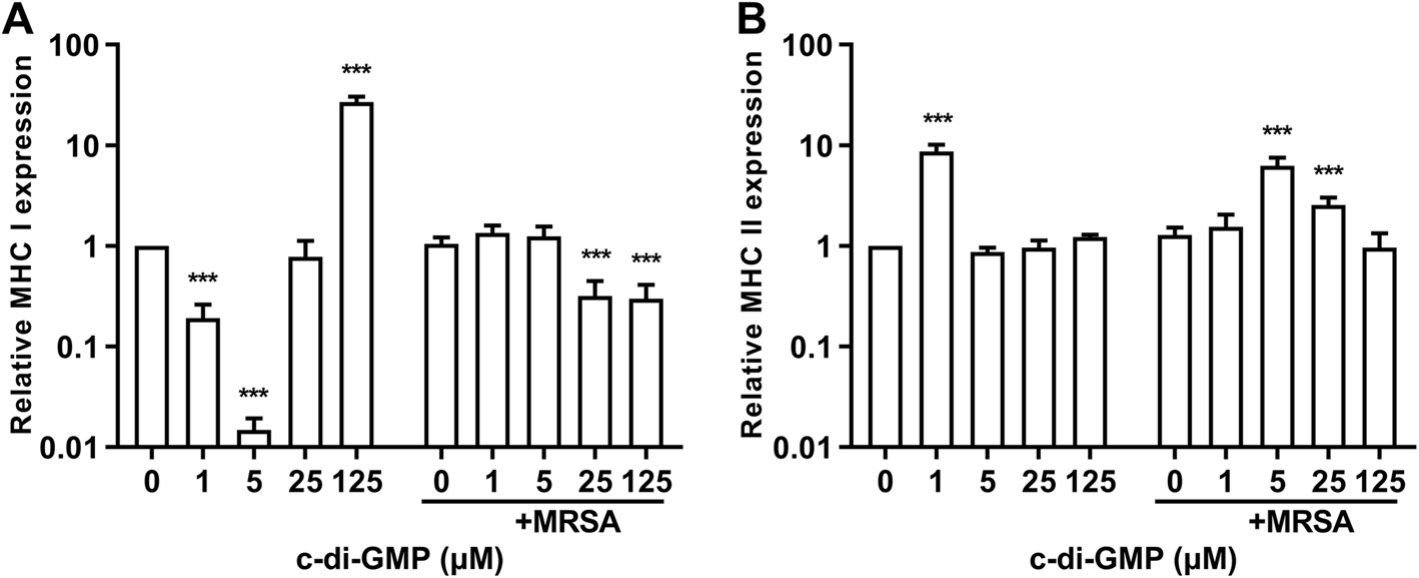
Stimulation of HaCaT cells with c-di-GMP induces gene expression of MHC class I and II. HaCaT cells were incubated for 12 h with c-di-GMP at 0, 1, 5, 25, or 125 μM and challenged with MRSA strain HRCH2019-1 for 3 h. Infected and uninfected control cells was harvested and total RNA was extracted with TRIzol. Relative gene expression of MHC class I and II was determined by quantitative real-time PCR following the 2.^−ΔΔCt^ method with GAPDH expression for normalization. **A** Relative gene expression of MHC class I. **B** Relative gene expression of MHC class II. Significant differences compared with 0 μM c-di-GMP for both the MRSA-challenged and unchallenged cells were identified by two-way ANOVA with Dunnett’s multiple comparisons test (GraphPad Prism). ***, *P* < 0.001

Keratinocytes not only participate in innate immunity, but also act as a non-canonical antigen-presenting cell (APC) to activate adaptive immunity [34]. Since c-di-GMP has previously displayed adjuvant-like stimulatory activities and the ability to induce MHC class I and II expression in immune cells [21, 35, 36], the effect of c-di-GMP on MHC class I and II gene expression was investigated for HaCaT cells in the presence and absence of MRSA strain HRCH2019-1. In the absence of MRSA, gene expression of MHC class I was highly induced by c-di-GMP at the highest dose of 125 μM (27-fold), while at the lower c-di-GMP doses expression seemed to be significantly repressed (Fig. 3A). Importantly, in the presence of MRSA strain HRCH2019-1, MHC class I gene expression was actually repressed at the higher c-di-GMP doses (25 and 125 μM), indicating that MRSA displays the ability to override c-di-GMP-mediated activation of MHC class I gene expression in HaCaT cells. In contrast, c-di-GMP induced gene expression of MHC class II ninefold at the lowest dose (1 μM) only, while in the presence of MRSA expression was induced sixfold at 5 μM and 2.5-fold at 25 μM (Fig. 3B), indicating that MRSA strain HRCH2019-1 was not able to inhibit c-di-GMP-mediated activation of MHC class II.

### C-di-GMP inhibits MRSA in a murine skin wound infection model

**Fig. 4:**
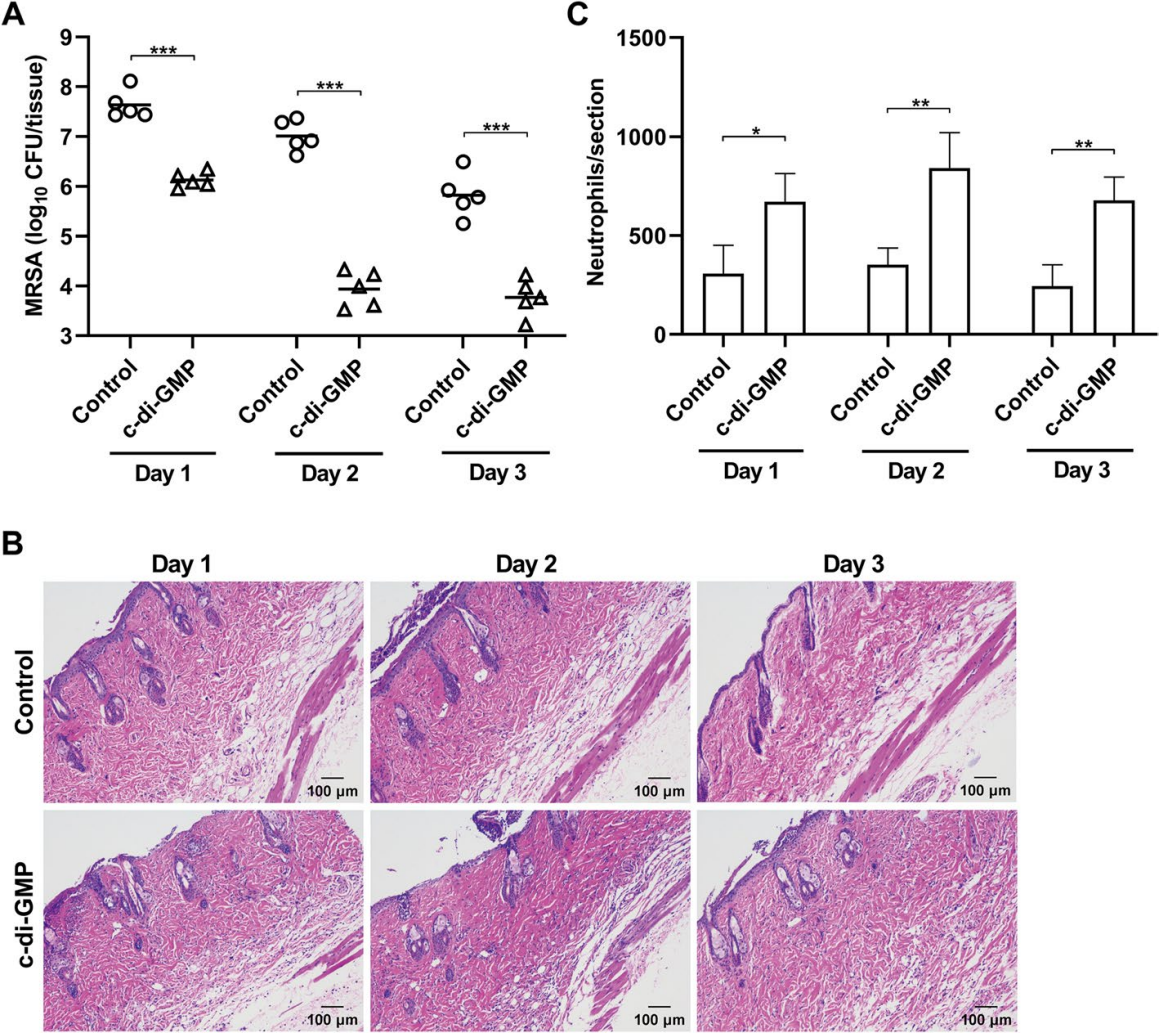
Pre-treatment with c-di-GMP attenuates MRSA colonization in a murine skin wound infection model. Groups of mice were pre-treated with 200 nmol c-di-GMP or PBS and 24 h later damaged skin sections were challenged with MRSA strain HRCH2019-1. Challenged skin sections were excised for 3 consecutive days post infection for quantification of bacterial CFU counts and histology analysis after H&E staining. **A** Quantification of MRSA CFUs from challenged skin tissue sections. Significant differences between the control and c-di-GMP pre-treatment group were identified by two-way ANOVA with Sidak’s multiple comparisons test (GraphPad Prism). ***, *P* < 0.001. **B** Representative microscopy images of MRSA-challenged H&E-stained skin tissue sections. **C** Quantification of neutrophils in MRSA-challenged H&E-stained skin tissue sections. Significant differences between the control and c-di-GMP pre-treatment group were identified by two-way ANOVA with Sidak’s multiple comparisons test (GraphPad Prism). *, *P* < 0.05; **, *P* < 0.01

The in vivo anti-MRSA activity of c-di-GMP was investigated in a murine skin wound infection model. Mice pre-treated with c-di-GMP showed a major reduction in bacterial loads on the infected skin sections compared with untreated control animals (Fig. 4A). One day after colonization, bacterial CFU counts were 37-fold lower in the c-di-GMP-treated animal compared with control animals, which further improved to 1,100-fold reduced CFU counts on day 2 and 130-fold reduced CFU counts on day 3. Further analysis of the pathology of H&E-stained tissue sections revealed a mild to moderate degree of inflammatory cell infiltration, and the presence of neutrophils appeared more prominent in c-di-GMP-treated mice than in the PBS-treated control mice (Fig. 4B). Indeed, quantification of neutrophils in the skin tissue sections showed that neutrophil infiltration was significantly enhanced in c-di-GMP-treated mice (Fig. 4C). The tissue sections did not show clear signs of tissue damage as a result inflammatory cell infiltration for both the control and c-di-GMP-treated mice. Therefore, these animal experiments indicated that c-di-GMP stimulates an anti-MRSA immune response, which enhanced clearance of MRSA strain HRCH2019-1 in an infection model.

## Discussion

Antimicrobial therapeutic options for treatment of multidrug-resistant MRSA infections have become increasingly limited, while the pipeline for delivery of novel antibiotics has not been forthcoming [37]. Therefore, strategies that stimulate immune-based eradication of MRSA have been suggested as alternative to conventional antimicrobials [14]. The second messenger c-di-GMP, which is unique to bacteria and not produced by eukaryotes, has previously displayed the ability to stimulate strong innate and adaptive immune responses. It is basically a chemotactic molecule that is sensed in the host cell cytosol by STING and NLRP3, which subsequently induce type I IFN and IL-1β responses, respectively [18–20]. These responses recruit monocytes and granulocytes and stimulate maturation of DCs and T cells [21]. Based on these qualities, c-di-GMP has been included in numerous vaccine studies as an adjuvant, and displayed the ability to raise antigen-specific immune responses with antibacterial, antiviral and anti-tumor activities [21, 24, 38–42]. Importantly, it has previously been shown that keratinocytes, which play an essential role in resisting *S. aureus* during a skin infection, are able to sense DNA molecules like c-di-GMP in their cytoplasm and induce STING and innate immune responses [43]. Upon exposure to *S. aureus*, keratinocytes have previously displayed the ability to secrete antimicrobial peptides [21, 44] and the neutrophil attracting chemokines CXCL-1, CXCL-2 and CXCL-8 [31], the latter mediated by IL-1 and IL-17 [30, 31, 45–48]. In addition, keratinocytes express various Toll-like receptors for sensing of bacterial pathogens and mounting innate responses [49–52]. Particularly, it has been shown for keratinocytes that TLR-2 senses *S. aureus* and activates NF-κB responses [53] and IL-1α-dependent secretion of CXCL-1, CXCL-2 and CXCL-8 [31]. In our current study, we showed that colonization of HaCaT cells by MRSA strain HRCH2019-1 was reduced by stimulation with c-di-GMP in a dose-dependent manner. HaCaT cells challenged with MRSA strain HRCH2019-1 showed induced secretion of four tested antimicrobial peptides hBD-1, hBD-2, hBD-3 and LL-37, but induction of hBD-1 was most pronounced and responsive to c-di-GMP stimulation, while hBD-2 and LL-37 were induced to a lesser extent. In contrast, it has been shown that keratinocytes cultured from patients display enhanced killing activity of *S. aureus* compared with HaCaT cells, and killing appeared largely dependent of hBD-3, because blocking of hBD-3 activity inhibited the keratinocyte killing activity [29]. Similarly, hBD-3 showed the strongest in vitro killing activity against *S. aureus*, followed by LL-37 and hBD-2, while no killing activity was detected for hBD-1 [29]. Therefore, the functionality of the strong hBD-1 response we observed in HaCaT cells upon exposure to MRSA and c-di-GMP stimulation is doubtful and killing activity was likely provided by the other antimicrobial peptides.

Although neutrophil recruitment is a hallmark of an *S. aureus* skin infection, it has several mechanisms aimed at inhibiting the recruitment of neutrophils to the site of infection [54–56]. Our analysis on the secretion neutrophil-attracting chemokines by HaCaT cells upon exposure to MRSA and c-di-GMP also indicated that secretion of CXCL-1 and CXCL-8 was reduced by MRSA activity. However, stimulation of HaCaT cells with lower doses of c-di-GMP enhanced secretion of these chemokines and counteracted the inhibitory activity of MRSA. This positive activity was likely also responsible for the elevated influx of neutrophils observed after c-di-GMP pre-treatment in the mouse skin wound infection model compared with untreated mice. Although strong influx of inflammatory cells and such as neutrophils might pose an increased risk for inflammatory tissue damage or immune related adverse events, neutrophils have also been associated with tissue protection and repair [57, 58]. Furthermore, tissue sections for both c-di-GMP-stimulated and control mice did not show obvious tissue damage. Most importantly, pre-treatment with c-di-GMP resulted in accelerated clearance of MRSA strain HRCH2019-1 from the infection model. On day 2 after infection, bacterial loads were over 1,000-fold lower in the c-di-GMP pre-treated mice compared with untreated controls. Similar results were previously demonstrated for ci-di-GMP treated mice in an *S. aureus* mastitis infection model, which displayed over 10,000-fold reduced CFU counts in mammary glands compared with untreated controls [21, 23]. These data indicate that c-di-GMP is a potent immuno-stimulating agent with the ability to induce responses equipped at targeting *S. aureus*. However, these infection models also highlighted that *S. aureus* was not fully eradicated by c-di-GMP treatment, since significant *S. aureus* fraction remained viable for the full during of these in vivo experiments. This limitation does not appear to be specific for *S. aureus*, since similar results were obtained in studies investigating the immuno-stimulating antimicrobial activity of c-di-GMP against several bacterial respiratory pathogens [21, 25, 26, 28]. For a pertussis mouse infection model, it was shown that c-di-GMP pre-treatment reduced *B*. *pertussis* CFU counts from lung tissues by up to 1,000-fold over a six-day period compared with control mice [26]. However, despite this major reduction, bacteria were not fully eradicated and a large fraction of surviving bacteria remained present in lung tissues. Similarly, for a bacterial pneumonia mouse infection model, pre-treatment with c-di-GMP resulted in a fivefold reduction *K. pneumoniae* loads in the lungs compared with controls and a 1,000-fold reduction in CFU counts in the blood, but particularly in the lungs bacterial numbers remained above 10^5^ CFU [21]. Therefore, these studies provide compelling evidence of the potent in vivo immunomodulatory antimicrobial activity of c-di-GMP, which might be sufficient as prophylactic to prevent bacterial infections in high-risk patient populations such as patients with burn wounds. However, to obtain full eradication of bacterial pathogens, particularly when exposed to elevated doses, c-di-GMP should likely be combined with other antimicrobial agents.

There are some limitations to the current study that should be emphasized. All MRSA experiments were performed with a single recent clinical isolate. Therefore, results presented in this study might not fully reflect the natural diversity and responses observed for MRSA. Furthermore, this study exploited HaCaT cells for in vitro evaluation of the impact of c-di-GMP on MRSA colonization and secretion of AMPs and chemokines. HaCaT is a spontaneously immortalized human keratinocyte cell line that expresses all major surface markers and displays functional characteristics of primary keratinocytes [59, 60]. However, there are also functional differences between HaCaT cells and primary keratinocytes, for instance, primary keratinocytes express LL-37 upon exposure to lipopolysaccharides and ultraviolet B irradiation, while this response is not observed in HaCaT cells [61]. Furthermore, primary keratinocytes show stronger *S. aureus* killing activity compared with HaCaT cells, which is largely dependent on hBD-3 [29], while c-di-GMP stimulation did not further enhance hBD-3 secretion in MRSA-challenged HaCaT cells compared with unstimulated control cells. Finally, translation of the in vivo infection study performed in mice to therapeutic efficacy in humans is not straightforward, also given that presence, expression and regulation of AMPs and chemokines is distinct. For instance, mice lack a true CXCL8 homologue [62], which in humans is important for neutrophil recruitment [33].

In conclusion, in this study we demonstrated that c-di-GMP displays a potent immuno-stimulating activity on human epidermal keratinocytes, which enhanced secretion of antimicrobial peptides and chemokines upon stimulation, and subsequently decreased colonization by MRSA. Furthermore, MRSA colonization of c-di-GMP pre-treated mice was significantly inhibited in a skin wound infection model. Therefore, c-di-GMP might be a suitable alternative prophylactic or therapeutic immuno-modulating agent to prevent or treat MRSA infections in skin or burn wound patients.

## Materials and methods

### Preparation of c-di-GMP

C-di-GMP (Biolog Life Science Institute, Germany) was dissolved in sterile pyrogen-free water. Absence of the endotoxin lipopolysaccharide was verified using the Limulus Amebocyte Lysate Assay kit (Xiamen BioEndo Technology, China) according to manufacturer’s instructions. Solutions of c-di-GMP were subsequently used for stimulation or treatment of HaCaT cells or BALB/c mice.

### Culture and storage of MRSA clinical isolate

The MRSA strain used in this project was a clinical isolate provided by Dr. Shibiao Ding (Hangzhou Red Cross Hospital, Hangzhou, China). The MRSA strain, named HRCH2019-1, was isolated in 2019 from a male patient at the Respiratory and Critical Care Department and was identified as MRSA by VITEK 2 Compact 60 (BioMérieux) and displayed resistance to oxacillin, penicillin, clindamycin and erythromycin. MRSA strain HRCH2019-1 was stored at -80 °C in tryptic soy broth (TSB; Hope Biotechnology, China) containing 15% glycerol (Biosharp, China). For all experiments, overnight grown MRSA strain HRCH2019-1 cultured in TSB at 37 °C and 5% CO_2_ was diluted 1:50 in fresh TSB and cultured for 2 h to obtain log-phase homogeneous cultures. Bacterial cells were collected, washed twice with sterile PBS (Gibco, The Netherlands), and resuspended in sterile PBS at appropriate concentrations before use in cell culture and animal experiments.

### HaCaT cell culture, c-di-GMP stimulation, viability and MRSA colonization assays

HaCaT human epidermal keratinocyte cells, purchased from the Cell Bank of the Chinese Academy of Sciences (CAS, Shanghai, China), were cultured for all experiments in Dulbecco’s Modified Eagle’s Medium (DMEM; Gibco, The Netherlands) containing 10% Fetal Bovine Serum (FBS; Gibco, The Netherlands) and grown at 37 °C 5% CO_2_. For viability assays, HaCaT cells were seeded in 96-well plates (Corning, USA) at 5 × 10^3^ cells/well and grown for 24 h in cell culture medium containing c-di-GMP at final concentrations of 0, 1, 5, 25 or 125 μM. Cell viability was determined using the CCK-8 cell viability assay (Dojindo Laboratories, Japan) according to manufacturer’s instructions. For c-di-GMP stimulation and MRSA invasion assays, HaCaT cells were seeded in 6-well plates (Corning, USA) at 2 × 10^5^ cells/well and grown overnight. Cells were subsequently washed and grown for 12 h in fresh cell culture medium containing c-di-GMP at final concentrations of 0, 1, 5, 25 or 125 μM. Cells were challenged with MRSA strain HRCH2019-1 suspended in pre-warmed cell culture medium at a multiplicity of infection (MOI) of 20 and cells were incubated for 3 h. Challenged and uninfected control cells were subsequently harvested for determination of MRSA viability and extraction of total RNA, while cell culture medium was used for quantification of secreted cytokines and antimicrobial peptides by ELISA. For quantification of viable MRSA associated with the HaCaT cells, cells were lysed with 0.5% Triton X-100 (Sigma, USA) and samples were serial diluted and plates on tryptic soy agar (TSA; Hope Biotechnology, China). After incubation for 24 h at 37 °C and 5% CO_2_ colony forming units (CFUs) were enumerated.

### Quantification of cytokines and antimicrobial peptides by ELISA

The levels of the antimicrobial peptides (AMPs) human β-defensin 1 (hBD-1), hBD-2, hBD-3 and LL-37, and the chemokines CXCL1 and CXCL8 were determined in the HaCaT cell culture medium using corresponding ELISA kits (Cusabio, China), as specified by the manufacturer.

### RNA extraction, cDNA synthesis and quantitative reverse transcription polymerase chain reaction (qRT-PCR)

HaCaT cells were washed with 0.1% DEPC pre-treated ddH_2_O (Sigma, USA), followed by the addition of 1 ml RNAiso (Takara, Japan) directly into each well and total RNA extraction using TRIzol reagent (Takara, Japan) according to manufacturer’s protocols. Subsequent removal of gDNA and synthesis of cDNA was performed with the Prime Script RT reagent Kit with gDNA Eraser (Takara, Japan) following manufacturer’s protocols and cDNA was stored at -20 °C until usage. Finally, qRT-PCR was performed with TB Green Premix Ex Taq (Takara, Japan), cDNA and primers (MHC I-F: CCTACGACGGCAAGGATTAC; MHC I-R: TGCCAGGTCAGTGTGATCTC; MHC II-F: TCCTGCATGGCGACTCTGAC; MHC II-R: CTTCTCTTCCTGGCCGTTCC; GAPDH-F: TGAAGGTCCGGAGTCAACGGATTGGT; GAPDH-R: CATGTGGGCCATGAGGTCCACCAC) in 20-μl reactions on a Roche LightCycler 480II (Roche, Switzerland). Relative expression levels of the HaCaT cellular genes MHC I and MHC II were normalized according to GAPDH levels. Relative expression was determined following the 2^−ΔΔCt^ method.

### Murine skin wound MRSA infection model

All procedures followed the guidelines of Administration of Affairs Concerning Experimental Animals of the People’s Republic of China and are in adherence with ARRIVE guidelines. The animal studies and procedures were approved by the Zhejiang University Animal Care and Use Committee under project license number 2015–008-05. The animals lived under specific-pathogen-free conditions were given free access to sterile water and certified mouse chow. Female pathogen-free 6- to 8-week-old BALB/c mice (Nanjing Biomedical Research Institute, China) were used for all experiments. The murine skin wound infection experiments were performed as described previously [63–66], with minor modifications. A total of 30 mice were randomly divided into a treatment (c-di-GMP) and control group (PBS). The two groups were injected intraperitoneally with 200 nmol of c-di-GMP or PBS 24 h prior to the MRSA infection. Before the skin infection, mice were anesthetized by intraperitoneal injection of a xylazine-ketamine cocktail (100 mg/kg + 5 mg/kg) and the back fur was subsequently shaved using a sterile razor. An area of about 2 cm^2^ was tape-stripped 10 times with adhesive bandage (Tensoplast), which resulted in visible superficial skin damage. For each mouse, 2 separate sections of the damaged skin area were inoculated with 1 × 10^7^ CFU of MRSA strain HRCH2019-1. For both the treatment and control group, the infected skin areas were excised from the mice at 1, 2, and 3 days post infection (5 mice per group per day) and for each mouse one section was placed in 1 mL sterile PBS for homogenization and CFU quantification and one section in 10% formaldehyde solution for histomorphometric analysis. Skin section homogenates were serial diluted and plated onto TSA supplemented with 6 mg/L methicillin. CFUs were enumerated after 24 h incubation at 37 °C and 5% CO_2_.

### Histomorphometric analysis

Murine skin tissues were immersed in 10% neutral formaldehyde solution (pH7.4; Biosharp, China) for 48 h. Tissues were rinsed, dehydrated and embedded in paraffin and 4-µm thin sections were stained with hematoxylin and eosin (H&E) solution (Biosharp, China). Images of the H&E-stained tissue were captured on an Olympus CX41 microscope.

### Statistical analysis

Statistical analyses were performed using GraphPad Prism 8.0 (GraphPad Software, San Diego, USA). *P*-values were determined by two-tailed Student’s *t*-test. *P*-values < 0.05 were considered significant.

## Data Availability

All data generated or analysed during this study are included in this published article.

## Abbreviations

MRSA: Methicillin-resistant *Staphylococcus aureus*
c-di-GMP: Cyclic di-guanosine monophosphate
HaCaT: Human epidermal Keratinocytes
AMPs: Antimicrobial peptides
hBD: Antimicrobial peptides human β-defensin
CXCL: Chemokine (C-X-C motif) ligand protein
CFU: Colony-forming Units
STING: Stimulator of interferon genes
IFN: Interferon
NLRP3: NOD-like receptor protein 3
IL: Interleukin
DC: Dendritic cell
mSEC: Mutant staphylococcal enterotoxin C
ClfA: Clumping factor A
APC: Antigen-presenting cell
